# Bone outcomes in virally suppressed youth with HIV switching to tenofovir disoproxil fumarate

**DOI:** 10.4102/sajhivmed.v22i1.1243

**Published:** 2021-08-05

**Authors:** Kate Braithwaite, Tristan D. McPherson, Yanhan Shen, Stephen Arpadi, Stephanie Shiau, Gillian Sorour, Karl-Günter Technau, Michael T. Yin

**Affiliations:** 1 Department of Paediatrics and Child Health, Faculty of Health Sciences, Empilweni Services and Research Unit, Rahima Moosa Mother and Child Hospital, University of the Witwatersrand, Johannesburg, South Africa; 2 Department of Medicine, Division of Infectious Diseases, Vagelos College of Physicians and Surgeons, Columbia University Irving Medical Center, New York, United States of America; 3 G.H. Sergievsky Center, Vagelos College of Physicians and Surgeons, Columbia University Irving Medical Center, New York, United States of America; 4 Department of Pediatrics, Vagelos College of Physicians and Surgeons, Columbia University Irving Medical Centre, New York, United States of America; 5 Department of Biostatistics and Epidemiology, Rutgers School of Public Health, Rutgers University, Piscataway, New Jersey, United States of America

**Keywords:** renal, bone, adolescents, tenofovir disoproxil fumarate, South Africa

## Abstract

**Background:**

Tenofovir disoproxil fumarate (TDF) is included in first-line antiretroviral treatment (ART) for adolescents living with HIV (ALWH). Associated toxicities remain a concern.

**Objective:**

We evaluated bone and renal safety outcomes in virologically suppressed South African ALWH after switching to TDF.

**Method:**

We recruited virally suppressed (< 100 copies/mL) adolescents, aged 15–20 years, who switched from an abacavir (ABC)-based to a TDF-based efavirenz regimen. Bone mass and renal function were assessed at Week 0 and at Week 24 after the switch to TDF using dual-energy X-ray absorptiometry (DXA) and serum renal markers. A change in the lumbar spine (LS) and the whole-body less head (WBLH) bone mineral density (BMD) Z-scores and the estimated glomerular filtration rate (eGFR) between the two measures were compared (paired *t*-tests) and stratified by sex.

**Results:**

Fifty participants (48% male), with a median duration of prior ART of 11.4 years, were enrolled. Among 47 participants with 24-week DXA results, 15 (32%) had either no change or a decreased LS-BMD after the switch, with a mean change of –1.6%. Overall, more female participants experienced this outcome: 58% versus 4%, *P* < 0.0001. The mean change (standard deviation) in the LS-Z-score was –0.03 (0.25) and in the WBLH-Z-score was 0.02 (0.24). A decrease in the eGFR from 132.2 to 120.4 was observed (*P* = 0.0003); however, the levels remained clinically acceptable.

**Conclusion:**

South African ALWH switching from abacavir to TDF-based ART experienced statistically significant decreases in eGFR but not in LS and WBLH BMD. Female ALWH were more likely to experience a decrease in LS-BMD and may require closer monitoring.

## Introduction

Low bone mineral density (BMD) and increased fracture risk have been reported in persons living with HIV (PLWH), particularly older men and postmenopausal women.^1,2^ Decreased bone mass has also been reported in children, adolescents and young adults who acquire HIV early in life by either perinatal or sexual transmission.^3,4,5^ The 2019 World Health Organization (WHO) Update of Recommendations on First- and Second-Line Antiretroviral Treatment (ART) indicates that the preferred antiretroviral (ARV) regimen for 10–19-year-old adolescents living with HIV (ALWH) is tenofovir disoproxil fumarate (TDF) + lamivudine/emtricitabine (3TC/FTC) as anchor and, barring exceptional situations, either dolutegravir or low-dose efavirenz (EFV).^6^ Tenofovir disoproxil fumarate has excellent durability, high efficacy and pharmacokinetics that allow for once-daily dosing.^7,8,9^ Nevertheless, concern over the bone and renal toxicity of TDF, such as a decrease in BMD and glomerular and renal tubular dysfunction, persists. Some,^10,11,12,13^ but not all,^14,15,16,17^ studies in children report a decline in BMD. Tenofovir alafenamide (TAF), currently unregistered in South Africa (SA), may offer a bone-friendly alternative. Few studies of bone and renal outcomes among TDF-treated children and adolescents exist, especially in sub-Saharan Africa (SSA), where comorbid conditions such as malnutrition, acute and chronic infectious diseases and other adverse environmental conditions are prevalent. The overall objective of this study is to evaluate bone and renal safety after a switch from abacavir (ABC) + 3TC + EFV to the fixed-dose combination (FDC) of TDF + FTC + EFV in clinically stable and virologically suppressed South African ALWH.

## Methods and materials

This was a prospective longitudinal study to assess the bone and renal effects of a switch to a TDF-containing ARV regimen in virally controlled, ARV-experienced ALWH.

### Study participants and sampling

**FIGURE 1:**
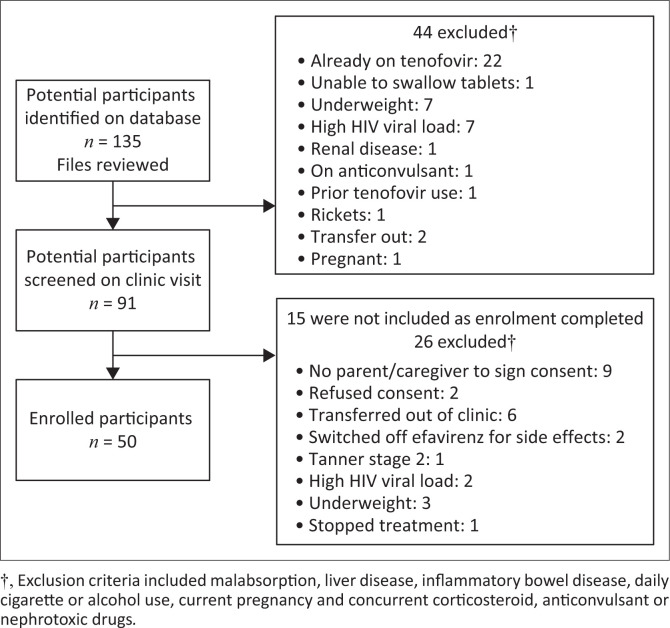
Study flow-chart of inclusion and exclusions of adolescents assessed for switch to TDF-containing ART.

Adolescents living with HIV were recruited from among peers receiving the usual medical care and treatment at Empilweni Clinic of the Rahima Moosa Mother and Child Hospital (RMMCH), Johannesburg, South Africa. Participants were selected through a sample of convenience. We identified all potential participants through the clinic database, conducted file reviews and screened eligible participants at their next scheduled appointment (Figure 1). Informed consent was obtained from caregivers and assent was obtained from participants prior to the start of study procedures.

Fifty adolescents were enrolled onto our study. Preliminary data from retrospective dual-energy X-ray absorptiometry (DXA) scans performed at the RMMCH on 25 ALWH indicated a 0.32 change (standard deviation [s.d.]: 0.9) in bone accrual over 12 months. Based on this, and assuming an s.d. of 0.9, 80% power and an alpha of 5%, we expected to detect an effect size of 0.36 with paired *t*-tests and 50 subjects. The participants who met the enrolment criteria were between 15 and 20 years of age, had an HIV-1 viral load (VL) of < 100 copies/mL within a 3-month period prior to the initial enrolment visit, weighed ≥ 40 kg and were Tanner stages 4 or 5. An HIV-1 VL of < 100 copies/mL was chosen, as this is the usual clinic cut-off required to switch from an ABC-based to a TDF-based FDC regimen, namely TDF (300 mg) + FTC (200 mg) + EFV (600 mg). Additional criteria included an estimated glomerular filtration rate (eGFR) of ≥ 80 mL/min as per South African national guidelines.^18^ The exclusion criteria are outlined in Figure 1.

### Methods and equipment

The study consisted of two visits: Week 0 and Week 24. At Week 0, participants were switched from ABC + 3TC + EFV to the FDC of TDF + FTC + EFV. From other studies, it is evident that the majority of the change in BMD occurs within the first 24 weeks after initiation of TDF.^13,19^ Therefore, we chose 24 weeks as our primary end point. Week 0 visits were performed between 29 November 2017 and 24 October 2018, and Week 24 visits were performed between 25 May 2018 and 17 April 2019.

At the Week 0 visit, participants were given a questionnaire to complete. A file review provided demographic information (age, sex, race or ethnicity), anthropometric measures (weight, height, body mass index [BMI]), a detailed ARV history, vitamin intake, lifestyle choices, a fracture history and the diagnosis of comorbid conditions (current or treated). Participants used a self-assessment tool to define their Tanner stage.^20^ A CD4 count was performed if there was no clinic result in the preceding three months. The BMI-for-age and height-for-age Z-scores were assessed using the WHO’s standard growth charts.^21^

Additional study assessments were performed at both visits: DXA of the whole-body less-head (WBLH) and the lumbar spine (LS) for BMD and bone mineral content (BMC) on a single Hologic densitometer with Apex software version 5.6.05 (Hologic Inc., Bedford, Massachusetts, United States). The bone area-1 size is the scanned area of the bone. The BMD is calculated by dividing the BMC by the bone area-1 size. The precision for BMD and BMC was < 1% for spine and < 2.5% for whole-body phantoms. The BMD and BMC measures are expressed as absolute values and as Z-scores from normative data provided by the Hologic database. Low BMD was defined as a Z-score of ≤ –2.0 on either WBLH or LS. Poor bone outcomes at Week 24 were demonstrated by either no change or a decrease in the BMD Z-score. No change in the BMD Z-score was included in the assessment, as an increase in BMD Z-score is expected in this age group.

Blood and urine specimens were collected to check the bone turnover markers and renal function. The bone turnover markers included procollagen type 1 amino-terminal propeptide (P1NP) and the C-terminal telopeptide of type 1 collagen (CTx), which were collected at both study visits and run at one time point to avoid batch effects. The renal markers were serum creatinine, serum urea nitrogen, serum phosphate, serum albumin, urine β-2-microglobulin (β2MG), urine microalbumin, urine phosphate and urine creatinine. These tests were all run in real time. The urine protein:creatinine ratio, urine β2MG creatinine ratio and fractional excretion of phosphate were calculated from these values. The eGFR was calculated using the revised Schwartz equation (2009). According to the South African guidelines at the time of this study, adolescents could be switched to TDF if the eGFR was ≥ 80 mL/min and had to be taken off TDF if the eGFR was < 50 mL/min.^18^ The HIV VL was repeated at Week 24. An undetectable VL was defined as < 50 copies/mL.

### Statistical methods

Descriptive statistics were used to characterise the participants at the time of enrolment. Numeric changes in outcomes from Week 0 to Week 24 were calculated, and Shapiro–Wilk tests were conducted to decide if continuous variables were normally distributed. Student’s *t*-tests using means and Wilcoxon’s rank-sum tests using medians were conducted for normally distributed and non-normally distributed measurements, respectively.

Fisher’s exact tests were used to compare the proportions of poor bone outcome. Univariable logistic regression was used to determine the possible factors associated with poor bone outcome: demographic characteristics, anthropometric measures, ARV uptake, VL, CD4 and changes of bone turnover markers. All *P*-values were two-tailed, and *P*-values of < 0.05 were considered statistically significant. In addition, 95% confidence intervals (CIs) were calculated. Finally, SAS version 9.4 (SAS Institute Inc., Cary, North Carolina, United States) was used for all statistical analyses.

### Ethical considerations

Ethical approval was obtained from the University of the Witwatersrand Human Research Ethics Committee (medical approval number: M161107) and the Columbia University Institutional Review Board prior to enrolment of participants.

## Results

One participant was included based on a point-of-care (Xpert HIV-1 quantitative assay; Cepheid, Sunnyvale, California, United States) HIV VL result of < 40 copies/mL; however, the formal VL done at the laboratory was 128 copies/mL. The results were calculated including and excluding this participant, and no significant differences were observed. The results described below include this participant.

### Demographics at time of switch to TDF

All participants had complete Week 0 information. Twenty-four (48%) were male, with a median age of 15.5 years (interquartile range [IQR]: 15.1–16.1). While we included participants aged 15–20 years, most were in the younger age range as adolescents in the clinic are switched to the TDF + FTC + EFV FDC as soon as they are eligible. All participants were perinatally HIV infected, with a median duration of ARV use of 11.4 years (IQR: 7.1–13.0). Six (12%) had a prior AIDS-defining illness. The median CD4 count was 728 cells/µL (IQR: 543–1017), and 39 (78%) had an undetectable VL at the Week 0 visit. Nine (18%) had a height-for-age Z-score of ≤ –2 and on BMI, three (6%) were classified as thin and five (10%) as overweight. No participant reported a family history of hip fractures, although six (12%) reported a previous trauma-related fracture (Table 1).

**TABLE 1:** Demographics for 50 virologically controlled ALWH switching to TDF.

Week 0 demographics	Statistic					
	Median	IQR	*n*	%	Mean	s.d.
Age (years)	15.5	15.1–16.1	-	-	-	-
Weight (kg)	48.6	42.6–54.2	-	-	-	-
Height (cm)	158.9	154.0–163.0	-	-	-	-
BMI (kg/m^2^)	19.7	3.6	-	-	-	-
**Sex**						
Male	-	-	24	48	-	-
Female	-	-	26	52	-	-
**Tanner stage**						
4	-	-	37	74	-	-
5	-	-	13	26	-	-
**Prior or occasional tobacco use**						
Yes	-	-	3	6	-	-
No	-	-	47	94	-	-
**Prior or occasional alcohol use**						
Yes	-	-	3	6	-	-
No	-	-	47	94	-	-
**Prior substance use**						
Yes	-	-	1	2	-	-
No	-	-	49	98	-	-
**Fractures**						
Yes	-	-	6	12	-	-
No	-	-	44	88	-	-
**Multivitamin use**						
Yes	-	-	24	48	-	-
No	-	-	26	52	-	-
**AIDS**						
Candidiasis	-	-	1	2	-	-
Encephalopathy	-	-	3	6	-	-
Tuberculosis (disseminated)	-	-	1	2	-	-
Wasting syndrome	-	-	1	2	-	-
No	-	-	44	88	-	-
**Viral load within 3 months (copies/mL)**						
Detectable	-	-	11	22	-	-
Undetectable	-	-	39	78	-	-
Nadir CD4 count (cells/*µ*L)	-	-	-	-	344	232
Week 0 CD4 count (cells/*µ*L)	728	543–1017	-	-	-	-
Duration of antiretroviral use (years)	11.4	7.1–13.0	-	-	-	-

ALWH, adolescents living with HIV; BMI, body mass index; IQR, interquartile range; s.d., standard deviation; TDF, tenofovir disoproxil fumarate.

### Bone parameters

Several follow-up DXA results were excluded because of technical challenges. Consequently, only 47 (94%) participants had 24-week DXA-LS results and 46 (92%) had 24-week DXA-WBLH results. The DXA data were analysed for participants who had at least one DXA result at both time points. At Week 0, the prevalence of low BMD was 13 (28%) for LS and 12 (26%) for WBLH. The median LS and WBLH-Z-scores were –1.15 (IQR: –2.3 to –0.3) and –1.05 (IQR: –2.0 to –0.3), respectively. The mean changes (s.d.) in the LS and WBLH-Z-scores were –0.03 (0.25) and 0.02 (0.24), respectively. No participant had a decrease in the LS-Z-score from > –2.0 to ≤ –2.0, but one participant had this outcome for the WBLH-Z-score.

There was a statistically significant increase in the mean LS and WBLH bone area-1 size, BMC and BMD at Week 24 (Table 2). At Week 24, 15 (32%) participants demonstrated a decrease in LS-BMD, with a mean change of –1.6%. Of this group, 14 (93%) were female. Overall, a greater proportion of female than male participants had decreases in LS-BMD (13 [58%] vs 1 [4%], *P* < 0.0001; 95% CI: 3.5–267.6). At Week 24, 14 (30%) participants demonstrated decreases in WBLH BMD, with a mean change of –1.1%. However, there was no significant difference in reduction of WBLH BMD between female and male participants. There was no difference in the age of those with decreased or unchanged BMD and those with increased BMD. There were no significant associations between poor bone outcomes and the Week 0 weight, Week 0 CD4 count, nadir CD4 count, eGFR or urine protein/creatinine ratio (Table 3). No significant changes in CTx or P1NP were observed (Table 2).

**TABLE 2:** Bone parameters at Week 0 and Week 24.

Measurement	Week 0				Week 24				Change (s.d.)				*P*
	Mean	s.d.	Median	IQR	Mean	s.d.	Median	IQR	Mean	s.d.	Median	IQR	
**Bone mass (*n* = 47 for LS; *n* = 46 for WBLH)**													
LS BMC (g)	44.5	11.1	-	-	45.8	10.7	-	-	1.25	1.85	-	-	< 0.01
LS area (cm^2^)	52.1	7.5	-	-	53.1	7.4	-	-	0.98	1.74	-	-	< 0.01
LS-BMD (g/cm^2^)	0.85	0.14	-	-	0.86	0.13	-	-	0.01	0.02	-	-	0.01
LS-Z-score	−1.22	1.36	-	-	−1.24	1.30	-	-	−0.03	0.25	-	-	0.48
WBLH BMC (g)	-	-	1182	1038–1396	-	-	1256	1116–1468	-	-	68	111.9	0.23
WBLH area (cm^2^)	-	-	1392	1288–1534	-	-	1423	1327–1558	-	-	49.9	64.1	0.15
WBLH BMD (g/cm^2^)	0.87	0.09	-	-	0.88	0.09	-	-	0.01	0.03	-	-	< 0.01
WBLH-Z-score	−1.02	1.09	-	-	−0.99	1.08	-	-	0.02	0.24	-	-	0.5
**Bone turnover markers (*n* = 50)**													
CTx (ng/mL)	-	-	0.97	0.52–1.61	-	-	0.79	0.52–1.69	-	-	−0.03	0.46	0.73
P1NP (ng/mL)	-	-	175.8	101.0–250.0	-	-	154.0	84.2–250.0	-	-	−6.8	56.6	0.60

BMD, bone mineral density; BMC, bone mineral content; CTx, C-terminal telopeptide of type 1 collagen; IQR, interquartile range; LS, lumbar spine; P1NP, procollagen type 1 amino-terminal propeptide; s.d., standard deviation; WBLH, whole-body less head.

**TABLE 3:** Univariate logistic regression modelling associations between outcomes (decrease/no change in LS and WBLH BMD) and clinical and laboratory parameters.

Clinical/laboratory parameters	Decrease/no change in LS			Decrease/no change in WBLH		
	Odds ratio	95% CI	*P*	Odds ratio	95% CI	*P*
Female sex (ref: male)	30.8	3.5–267.6	< 0.01	2.040	0.559–7.448	0.28
Weight (kg)	1.011	0.932–1.098	0.79	1.067	0.979–1.162	0.14
Multivitamin usage: no (ref: yes)	0.772	0.226–2.639	0.68	0.662	0.187–2.347	0.52
VL detected (ref: undetected)	0.833	0.229–3.028	0.78	1.432	0.396–5.178	0.58
Week 0 CD4 count (cells/µL)	1.000	0.998–1.003	0.87	0.999	0.996–1.001	0.30
Nadir CD4 count (cells/µL)	0.999	0.997–1.002	0.62	1.000	0.998–1.003	0.77
Use of ART duration (years)	0.968	0.796–1.177	0.75	1.105	0.896–1.363	0.35
Urine PCR (mg/mmol)	1.861	1.047–3.308	0.03	0.714	0.376–1.356	0.30
Urine PCR change (mg/mmol)	1.027	0.840–1.255	0.80	1.189	0.908–1.556	0.21
eGFR (mL/min)	0.993	0.971–1.016	0.57	0.992	0.968–1.016	0.50
eGFR change (mL/min)	1.007	0.976–1.038	0.67	0.993	0.964–1.024	0.67
FePO_4_	1.036	0.995–1.080	0.09	0.986	0.946–1.028	0.52
FePO_4_ change	1.000	0.996–1.005	0.64	1.002	0.998–1.007	0.28
Baseline CTx (ng/mL)	0.123	0.026–0.573	0.01	0.599	0.235–1.527	0.28
CTx change (ng/mL)	1.007	0.997–1.018	0.17	0.985	0.969–1.002	0.09
Baseline P1NP (ng/mL)	0.983	0.973–0.993	< 0.01	0.991	0.982–0.999	0.03
P1NP change (ng/mL)	1.001	0.996–1.006	0.78	0.979	0.952–1.006	0.12

ART, antiretroviral treatment; BMD, bone mineral density; CI, confidence intervals; CTx, C-terminal telopeptide of type 1 collagen; eGFR, estimated glomerular filtration rate; FePO_4_, fractional excretion of phosphate; LS, lumbar spine; PCR, protein:creatinine ratio; P1NP, procollagen type 1 amino-terminal propeptide; VL, HIV viral load; WBLH, whole-body less head.

### Renal parameters

Two participants’ 24-week urine tests were uninterpretable because of menstruation. There were no significant changes in the fractional excretion of phosphate and the protein:creatinine ratio (Table 4). Statistically significant increases in serum creatinine were observed from 46.2 µmol/L to 50.7 µmol/L (*P* < 0.0001) and decreases in eGFR from 132.2 mL/min to 120.4 mL/min (*P* = 0.0003). The final levels, however, remained within clinically acceptable limits, and no participants had to be taken off TDF. Although adherence to medication was not specifically addressed, an HIV-1 VL of < 100 indicates good adherence. There was no significant change in HIV-1 VL over the 24 weeks (data not shown).

**TABLE 4:** Renal parameters at Week 0 and Week 24.

Measurement	Week 0				Week 24				Change (s.d.)				*P*
	Mean	s.d.	Median	IQR	Mean	s.d.	Median	IQR	Mean	s.d.	Median	IQR	
**Renal markers** †													
Serum creatinine (μmol/L)	46.2	10.2	-	-	50.7	10.2	-	-	4.5	6.4	-	-	< 0.0001
Serum urea nitrogen (mmol/L)	2.87	0.77	-	-	3.06	0.98	-	-	0.19	1.08	-	-	0.22
Serum PO_4_ (mmol/L)	1.35	0.21	-	-	1.38	0.26	-	-	0.03	0.23	-	-	0.33
Serum albumin (g/L)	45.6	2.5	-	-	45.9	2.9	-	-	0.24	3.30	-	-	0.61
Urine β2MG (mg/L)	-	-	0.08	0.03–0.14	-	-	0.08	0.05–0.19	-	-	0.03	0.11	0.29
Urine microalbumin (mg/L)	-	-	5.75	5.30–8.00	-	-	6.20	5.50–8.90	-	-	−1.4	9.41	0.28
Urine PO_4_ (mmol/L)	-	-	7.25	2.75–11.15	-	-	6.39	4.25–11.81	-	-	0.63	5.80	0.66
Urine creatinine (mmol/L)	-	-	8.25	4.45–11.43	-	-	8.04	5.38–12.88	-	-	0.48	5.64	0.49
**Calculated renal markers**													
Urine PCR (mg/mmol)	-	-	0.84	0.54–1.53	-	-	0.84	0.56–1.44	-	-	−0.34	1.31	0.96
eGFR (mL/min)	-	-	132.0	113.0–144.0	-	-	119.5	102.0–134.0	-	-	−11.9	21.6	0.03
Urine β2MG/creatinine ratio	-	-	0.010	0.007–0.013	-	-	0.011	0.008–0.014	-	-	0	0.01	0.40
FePO_4_	29.81	15.92	-	-	32.49	17.89	-	-	2.68	20.91	-	-	0.37

β2MG, β-2-microgloblin; eGFR, estimated glomerular filtration rate; FePO_4_, fractional excretion of phosphate; PCR, protein:creatinine ratio; PO_4_, phosphate; s.d., standard deviation; IQR, interquartile range.

† , *N* = 50; *n* = 48 for urine microalbumin and urine creatinine measurement.

## Discussion

South African ALWH on an EFV-based ART regimen who switched to TDF from other nucleoside reverse transcriptase inhibitors (NRTIs) experienced increases in LS and WBLH BMD over a 6-month period overall, consistent with a preserved trajectory of bone accrual. However, one-third of study participants had poor bone outcomes, with female participants significantly affected compared to male counterparts with regard to the LS-BMD.

There was also a small but statistically significant decrease in the eGFR in both female and male participants, of uncertain clinical significance.

Low BMD is a well-described finding in children and ALWH, with a higher prevalence in middle-income countries compared to resource-rich countries.^22,23^ The prevalence of low BMD in our study is similar to that of other middle-income countries, including Brazil (16.7% – 32.4%)^24,25^ and Thailand (24%).^26^ Multiple factors appear to be involved, including the effects of HIV-1 viral proteins, inflammatory cytokines and ART on bone cells and bone turnover.^27,28,29,30^ Exposure to these factors during childhood and adolescence, which are critical periods for skeletal growth, may impair the attainment of peak bone mass.^31^ This may have negative effects on adult bone mass and cortical and trabecular bone microarchitecture, which are major determinants of bone strength and fracture risk.^31^

Ninety per cent of peak bone mass is attained by the age of 18 years.^32^ Adolescents have an increased rate of bone accrual during puberty, which occurs earlier in girls. In boys this occurs between 14 years and 17 years and in girls between 11 years and 14 years; healthy female individuals have a decrease in the rate of bone accumulation from the age of 16 years.^33,34^ The median age of our participants was 15.5 years. More female than male participants experienced a decrease in LS-BMD, which may be associated with a decrease in bone accrual in girls at this age.

The independent effects of specific ARVs on BMD, either at ART initiation or upon switching, are sometimes difficult to ascertain because ARVs are given as combinations. An observed improvement in BMD may be the result of either a switch off an agent with negative effects on bone or the addition of an agent with a safer bone profile. Two studies that looked at a switch from stavudine (D4T) + 3TC + lopinavir/ritonavir to TDF + 3TC/FTC + EFV in virologically suppressed children and adolescents found no significant changes in BMD over 60 months^14^ and 10 years^35^ of follow-up. Aurpibul et al. found a significant decrease in BMD over 96 weeks in virologically suppressed children and adolescents who were on a non-nucleoside reverse transcriptase inhibitor-based regimen prior to switching to TDF + 3TC + EFV.^13^ Some of the difference in findings between the studies may be attributed to an improvement in BMD that may have occurred with a concurrent switch off protease inhibitors, cancelling the negative effects of TDF initiation.^4,14,36^ In this study, by maintaining EFV, and just switching the NRTI component, we were able to isolate the effects of switching to a single agent. However, these results may not be generalisable if the TDF switch occurs with a protease inhibitor as the backbone ARV.

Our study demonstrated that TDF initiation resulted in an overall increase in BMD among adolescents, which is the expected trajectory during growth; however, the effect differed between individuals and by sex, with a greater proportion of female participants experiencing net bone loss. Weight at the time of switch was not predictive of change in BMD, nor bone turnover marker levels. We also did not find an association between higher fractional excretion of phosphate or change in eGFR and bone loss. Our findings suggest that a switch to TDF may have negative consequences for specific individuals, especially girls; however, we cannot predict which individuals will experience greater bone loss based on clinical or laboratory parameters.

Tenofovir disoproxil fumarate-associated BMD loss is not well understood; however, one mechanism may be abnormal bone metabolism.^37^ Adult studies have demonstrated increased levels of CTx and P1NP in patients initiating ART irrespective of the regimen, but higher on TDF-containing ART.^38,39,40,41,42,43,44^ Levels increased in the first 24–48 weeks after ART initiation, and then decreased.^39,44^ Few studies have looked at bone biomarkers in children and adolescents. In Thai adolescents, TDF use was positively associated with CTx but negatively with P1NP when compared to non-TDF.^45^ This indicates abnormal rather than increased bone metabolism; this was not associated with low BMD in this study.^45^ There was no significant change in CTx or P1NP at 24 weeks after the switch to TDF, probably because our participants were ARV experienced and well suppressed at the time of the switch.

Another mechanism of TDF-associated bone loss is chronic phosphaturia and hypophosphataemia resulting from renal tubular dysfunction.^46^ There is little data on the renal effects of TDF on children and adolescents in SSA.

A cross-sectional study of Zimbabwean children and adolescents < 18 years and on TDF found hypophosphataemia, proteinuria and a decreased eGFR of < 90 mL/min per 1.73 m² in 11.1%, 32.8% and 35.9%, respectively.^47^ Proteinuria and a lower eGFR were associated with concomitant protease inhibitor (PI) use.^47^ It is difficult to compare studies, as participants in the Zimbabwean study were younger with a shorter duration of ART, and VL was not evaluated because of cost restraints. We found no evidence of hypophosphataemia, phosphaturia, proteinuria or renal impairment 24 weeks after the switch to TDF-containing ART. These findings are similar to other studies that have looked at virally suppressed children and adolescents switching to a TDF-containing regimen^13,48,49^; however, in a study extended to 132 weeks, 46% of patients developed mild or moderate hypophosphataemia.^15^ While most cases were mild and not clinically relevant,^15^ longer-term monitoring may be needed as this may contribute to low BMD over time.^46^

Currently in SA, adolescents from age 10 years who are suppressed on AZT/ABC/D4T + 3TC/FTC + EFV are being switched to TDF + 3TC/FTC with dolutegravir or high-dose EFV as an FDC after appropriate counselling.^50^ Our study was conducted when all eligible adolescents were switched to TDF + FTC + EFV from age 15 years, and it is therefore restricted to participants remaining on EFV. Integrase strand transfer inhibitor-based regimens with either TAF or ABC had minimal effects on BMD^51^; however, these regimens are not currently available in SA, either as FDCs or at all. While we advocate for a bone-sparing regimen in the adolescent population, TDF is likely to be used in SA and other low- and middle-income countries for the foreseeable future. Further research on the long-term effects of TDF in this population and younger adolescents from age 10 years is needed.

One limitation of our study is the lack of longitudinal data in healthy South African controls of similar age to determine whether the rate of bone accrual is similar to uninfected controls. Similarly, we lack data on the rate of change of BMD and BMC in ALWH who have not been switched to TDF-containing regimens.

Additionally, this was a small study of 50 participants, and because of technical difficulties, several patients did not have 24-week DXA results, which resulted in wide confidence intervals related to sex differences for LS-BMD. The significance of our results must be interpreted with this in mind. Furthermore, we have only 24 weeks of follow-up, which did not allow us to determine the long-term effects of TDF in this population, and the time interval between the DXA scans may have been too short to show changes in these older adolescents.^52^ However, other studies have shown decreasing BMD at 48 weeks and then a stabilisation of the BMD^11,13^; therefore, no significant changes in BMD at 24 weeks are promising. The Z-scores were not height adjusted, which may have overestimated the prevalence of low BMD at the time of the switch to TDF^45^; however, this does not affect our comparison between the two time points. Information on the participants’ diet, calcium intake and exercise was not included in this study, and so the effect of these factors on BMD changes could not be assessed.

In conclusion, we demonstrate that switching to TDF with an EFV-based regimen in virally suppressed ALWH results in overall gains in BMD commensurate with bone acquisition; however, more female than male participants experienced a 6-month decline in BMD and may require monitoring with DXA scans after the switch. Statistically significant reductions in eGFR were demonstrated 24 weeks after the switch to TDF, although eGFR was still clinically acceptable and well above the threshold at which guidelines recommend TDF withdrawal. Future research into the long-term effects of TDF on bone strength and renal function in this population, as well as in younger adolescents, is warranted.
